# Characteristics of mitosis in the gametophyte cells of the marine green alga *Monostroma angicava*

**DOI:** 10.1186/s40529-019-0256-z

**Published:** 2019-06-20

**Authors:** Yusuke Horinouchi, Tatsuya Togashi

**Affiliations:** Marine Biosystems Research Center, Chiba University, Kamogawa, 299-5502 Japan

**Keywords:** Chromosome number, Diurnal periodicity, Gametophyte, Metaphase arrest, Mitosis, Ulvophyceae

## Abstract

**Background:**

Some marine algae exhibit several characteristics of mitosis (e.g., the timing of mitosis such as diurnal periodicity) that are unique from those of land plants. Not only the timing but also other characteristics of mitosis, including the process itself and the number of chromosomes involved, are largely unknown in ulvophycean marine green algae. Effective mitotic inhibitors are useful for observing mitosis and identifying the number of chromosomes. However, few such inhibitors are available for ulvophycean algae. Here, we examined the timing and process of mitosis and the number of chromosomes with several mitotic inhibitors in the haploid gametophyte cells of the Ulvophyceae alga *Monostroma angicava*.

**Results:**

Mitosis did not occur during the light period but began immediately after the onset of the dark period. The typical process of mitosis was observed. The mitotic inhibitors colchicine and 8-hydroxyquinoline, which generally arrest mitosis in land plants, were ineffective in *M. angicava*. We found that three other mitotic inhibitors, amiprophos methyl, griseofulvin and oryzalin, are effective to arrest mitosis. With three-dimensional fluorescence microscopy, we demonstrated that there were nine chromosomes in each cell.

**Conclusions:**

In the gametophyte cells of *M. angicava*, mitosis occurs diurnally. It is triggered by the onset of the dark period. We identified the number of chromosomes as N = 9. Our study shows effective inhibitors to observe mitosis in ulvophycean algae.

**Electronic supplementary material:**

The online version of this article (10.1186/s40529-019-0256-z) contains supplementary material, which is available to authorized users.

## Background

Marine algae show several characteristic aspects of mitosis that are not observed in land plants. For example, regarding the timing of mitosis, their cell cycles often have diurnal periodicity, in which photosynthesis occurs during the daytime and mitosis occurs at night (e.g., Kuwano et al. 2014). In laminarialean brown algae, mitotic cell division becomes activated during the dark period (Makarov et al. 1995). A red alga, *Porphyra yezoensis*, also maximizes its mitotic activity at night (Oohusa 1980). In the green alga *Ulva compressa*, the onset of the dark period triggers mitotic cell division (Kuwano et al. 2008).

Some mitotic inhibitors that arrest mitosis in land plants, such as colchicine and 8-hydroxyquinoline (Evans et al. 1957; Wanner et al. 1991; Komaki and Schnittger 2016), might not take effect in algal species (e.g., McNaughton and Goff 1990). Mitosis is arrested at metaphase because these drugs inhibit the formation of microtubules (Komaki and Schnittger 2016). In contrast, other drugs such as amiprophos methyl (APM), griseofulvin and oryzalin have been used to inhibit microtubule polymerization in some algae (e.g., McNaughton and Goff 1990; Takahashi et al. 2001; Mine et al. 2011; Sommer et al. 2015). Mitotic inhibitors are useful for examining the details of the process of mitosis and counting the number of chromosomes.

There is limited understanding of the characteristics of mitosis, such as the timing and process of mitosis and the chromosome number, in ulvophycean marine green algae. This may be partly because observation of nuclei and identification of the chromosome number are difficult due to the small size of the nuclei and the lack of effective mitotic inhibitors. Details on the process of mitosis and the chromosome number have been reported only in a few species (e.g., Dube 1967; Yabu 1967; Aruga et al. 1996; Titlyanov et al. 1996; Zulkifly et al. 2013).

In the ulvophycean alga *Monostroma* *angicava*, little is known about mitosis, including the number of chromosomes involved. *M. angicava* has a heteromorphic haplo-diplontic life cycle with a multicellular haploid gametophyte and a unicellular diploid sporophyte (Tatewaki 1969; Horinouchi et al. 2019). The gametophytes are thalli of one-layered mononucleate somatic cells, and grow up to 15 cm high through mitosis. In this study, we examined the timing and detailed process of mitosis and the number of chromosomes with several mitotic inhibitors in *M. angicava*.

## Methods

### Material collection and culture

We collected a mature female gametophyte at Botofurinai, Muroran, Hokkaido, Japan (42°31′N, 140°98′E), in May 2013. Gametophytes of *M. angicava* release biflagellate gametes during spring at low tides (Togashi and Cox 2001). Gametes released from the gametophyte were separated from contaminants via their positive phototaxis (Togashi et al. 1999). The gametes were cultured under conditions described by Tatewaki (1969) with PES (Provasoli’s enriched sea-water) medium (Provasoli 1968) in culture chambers (LH-220S; NK System, Osaka, Japan). The gametes parthenogenetically developed into sporophytes at 14 °C under long-day conditions (14 h light:10 h dark cycle) created by cool white fluorescent lamps with an intensity of approximately 15 μmol photons/m^2^/s. The sporophytes released zoospores that developed into gametophytes at 12 °C under short-day conditions (10 h light:14 h dark cycle) created by cool white fluorescent lamps with an intensity of approximately 2.5 μmol photons/m^2^/s. The gametophytes were cultured at 10 °C under long-day conditions (14 h light:10 h dark cycle) created by cool white fluorescent lamps with an intensity of approximately 35 μmol photons/m^2^/s.

### Observations of somatic cell divisions

We fixed and decolored gametophytes 120 min, 360 min and 600 min after the onset of the light period and every 60 min after the onset of the dark period with a 3:1 ethanol:acetic acid solution at room temperature for 72 h. For more detailed examination of the timing of mitosis, we fixed and decolored the gametophytes every 10 min from 30 to 220 min after the onset of the dark period with a 3:1 ethanol:acetic acid solution. The fixed specimens were dealcoholized with distilled water for 15 min, stained with 5 µg/ml 4′-6-diamidino-2-phenylindole (DAPI) in VECTASHIELD mounting medium (Vector Laboratories, Burlingame, CA, USA) for 10 min and softly pressed with a glass coverslip. We observed the specimens using an IX81 fluorescent inverted microscope (Olympus, Tokyo, Japan) with a CCD camera (Olympus) and a Disk-Spinning Unit for Confocal Imaging (DSU). In this study, we defined dividing cells as cells with condensed chromosomes, because it is difficult to distinguish between nondividing cells and cells undergoing mitosis that lack condensed chromosomes (e.g., prophase cells). We measured the frequencies of dividing cells in a microscopic field of 5791 µm^2^ (n = 10 fields per measurement).

### Mitotic inhibitor treatments

From 60 to 180 min after the onset of the dark period, including the time when the cells had actively divided (see "Results" for more details), we treated the cells of young, growing *M. angicava* gametophytes with colchicine (0.05%, 0.1% or 0.5%), 8-hydroxyquinoline (2 mM), APM (1 µM, 5 µM, 10 µM or 50 µM), griseofulvin (1 µM, 5 µM, 10 µM or 50 µM) and oryzalin (1 µM, 5 µM, 10 µM or 50 µM) in PES medium in the culture chamber. Stock solutions of APM, griseofulvin and oryzalin were prepared with dimethylsulfoxide at 1 mM. They were diluted with PES medium. We then fixed and decolored them with a 3:1 ethanol:acetic acid solution. The specimens were dealcoholized with distilled water for 15 min, stained with 5 µg/ml DAPI in VECTASHIELD mounting medium for 10 min and softly pressed with a glass coverslip. We observed the specimens using an IX81 fluorescent inverted microscope. We measured the frequencies of dividing cells in a microscopic field of 5791 µm^2^ (n = 10 fields per measurement). The results were statistically analyzed with a 0.05 significance level with the Mann–Whitney U test using R version 3.2.3 (R Core Team 2015).

### Three-dimensional observation of chromosomes

We obtained cross-sectional images of chromosomes stained with 5 µg/ml DAPI every 0.1 µm using an IX81 fluorescence microscope with a DSU and constructed three-dimensional images using MetaMorph software (Molecular Devices, Tokyo, Japan). We counted the number of chromosomes based on three-dimensional images.

## Results

### Timing of mitosis

**Fig. 1:**
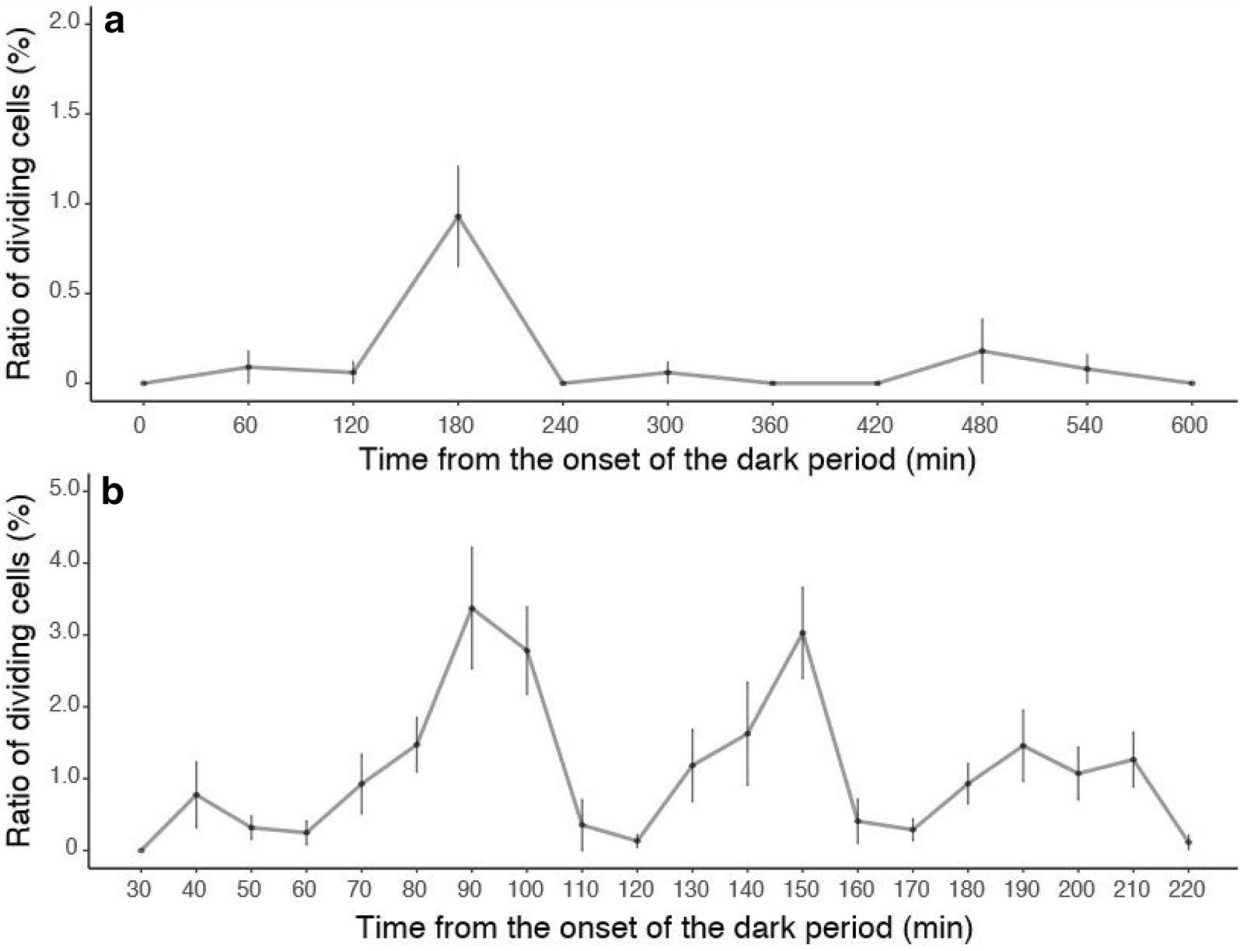
Ratio of dividing cells in gametophytes (mean and 95% confidence interval) after the onset of the dark period. **a** Ratio of dividing cells during the dark period examined every 60 min. **b** Ratio of dividing cells within 220 min from the onset of the dark period examined every 10 min

Although no dividing cells were observed during the light period, we found dividing cells with condensed chromosomes during the dark period (Fig. 1). In the long-term measurements every 60 min, dividing cells began to appear 60 min after the onset of the dark period (Fig. 1a). The ratio of dividing cells was the highest 180 min after the onset of the dark period. However, some dividing cells at different phases of mitosis were always observed during the dark period. In the short-term measurements every 10 min, we found that dividing cells began to appear 40 min after the onset of the dark period (Fig. 1b). The ratio of dividing cells was the highest 90 min after the onset of the dark period and was the second highest 150 min after the onset of the dark period.

### Process of mitosis

**Fig. 2:**
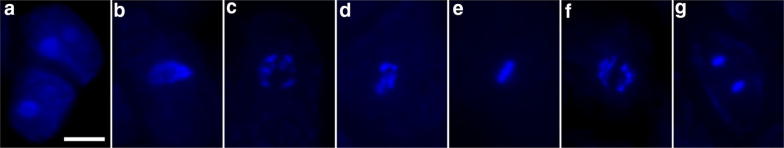
Process of mitosis in the gametophyte cells of *M. angicava*. **a** Interphase, **b** prophase, **c** early prometaphase, **d** late prometaphase, **e** metaphase, **f** anaphase, **g** telophase. Scale bar = 5 µm

Each phase of mitosis in the gametophyte cells of *M. angicava* is shown in Fig. 2. Nuclei were 3–4 µm in diameter at interphase (Fig. 2a) and swelled to 5–8 µm in diameter when the nucleoplasm become loose at prophase (Fig. 2b). Chromosomes gradually condensed and became observable at early prometaphase (Fig. 2c). Chromosomes loosely gathered with each other at late prometaphase (Fig. 2d). Chromosomes were densely assembled and arranged in a line at metaphase (Fig. 2e). Chromosomes became segregated and moved to the poles at anaphase (Fig. 2f). Chromosomes decondensed and became two daughter nuclei at telophase (Fig. 2g). We observed that somatic cells at different phases of mitosis simultaneously existed in each gametophyte.

### Mitotic inhibitor treatments

**Fig. 3:**
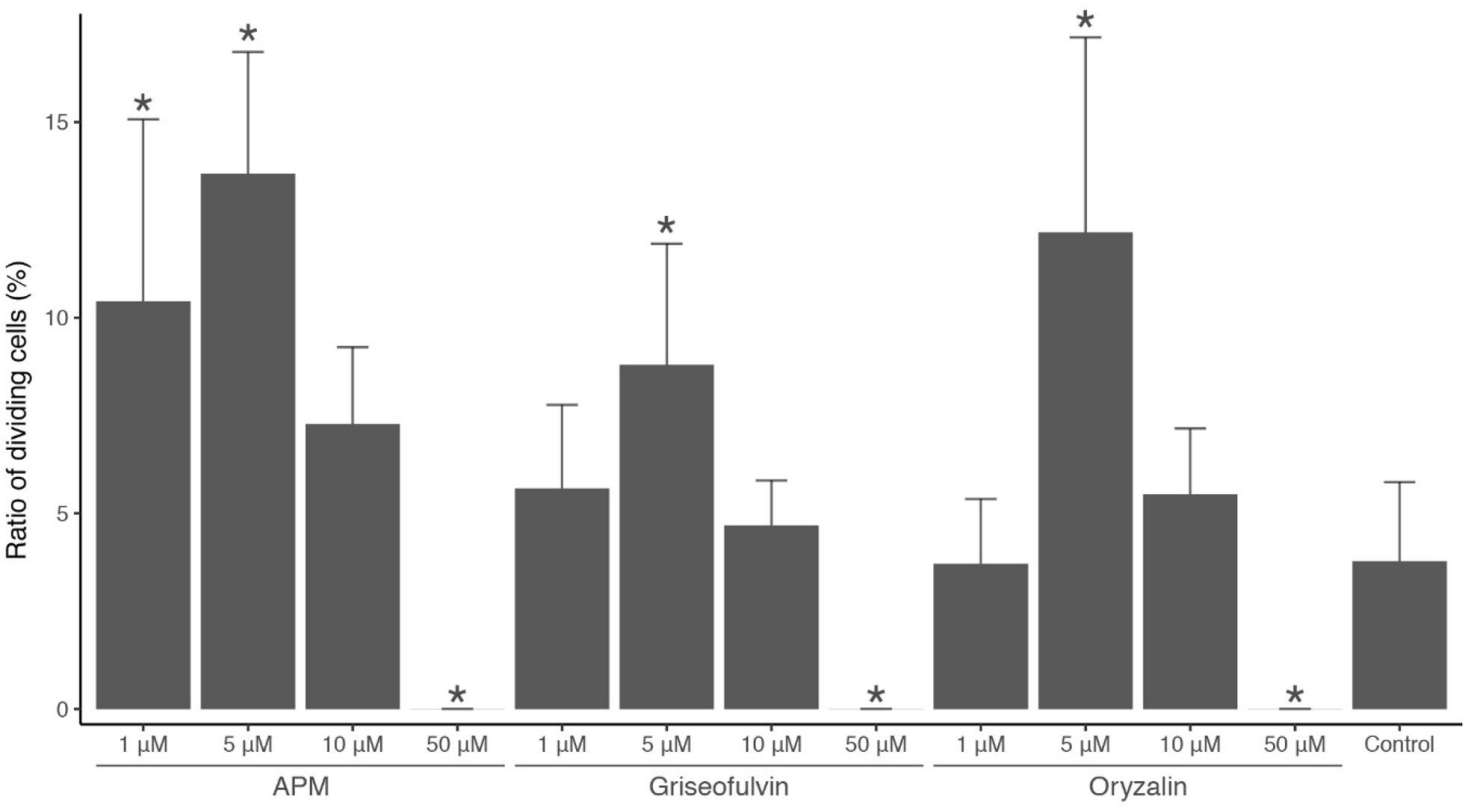
Ratio of dividing cells in gametophytes (mean and 95% confidence interval) after treatment with mitotic inhibitors. The asterisks indicate that the ratios are significantly different from that of the control (no treatments)

The ratios of dividing cells after treatment with 0.05%, 0.1% and 0.5% colchicine and 2 mM 8-hydroxyquinoline were not significantly different from that of the control group (no treatment) (p = 0.50, p = 0.50, p = 0.37 and p = 0.50, respectively). On the other hand, 1 µM and 5 µM APM, 5 µM griseofulvin and 5 µM oryzalin significantly increased the ratio of dividing cells (p = 0.01, p = 7.5 × 10^–4^, p = 0.03 and p = 0.01, respectively) (Fig. 3). With the high-concentration treatments (50 µM APM, griseofulvin and oryzalin), no dividing cells were observed (p = 7.5 × 10^–4^, p = 7.5 × 10^–4^ and p = 7.5 × 10^–4^, respectively) (Fig. 3). We found metaphase cells in these experiments.

### Three-dimensional observation of chromosomes

**Fig. 4:**
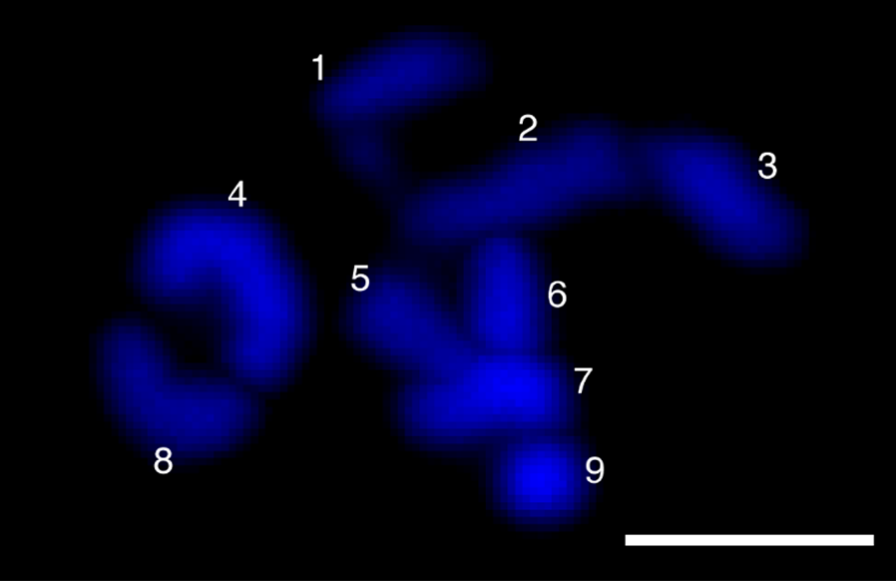
Number of chromosomes in the gametophyte cells of *M. angicava* (N = 9). The numbers indicate chromosomes. Scale bar = 2 µm. See also Additional file 1: Video S1

We obtained some clear three-dimensional images of the chromosomes (Additional file 1: Video S1) in late prometaphase cells (Fig. 2d), in which all the chromosomes were well condensed. The number of chromosomes in the haploid gametophyte cells of *M. angicava* was nine (Fig. 4, see also Additional file 1: Video S1).

## Discussion

Our observation revealed the timing of mitosis in the gametophyte cells of *M. angicava* (Fig. 1). We found no cells with condensed chromosomes during the light period (Fig. 1a). We began to observe cells with condensed chromosomes starting 40 min after the onset of the dark period (Fig. 1a). Therefore, the onset of the dark period seems to be the trigger of mitosis. Each cell division appeared to take approximately 50 min since the peaks in the ratio of dividing cells appeared approximately every 50 min (Fig. 1b). However, mitotic cell division was not perfectly synchronized, because dividing cells at different phases were always observed. It has been suggested that mitosis that occurs at night is useful for preventing irregular cell division because it temporally separates photosynthesis and cell division (Kuwano et al. 2014). Diurnal cell division, as observed in this alga, has been reported in several other multicellular marine algae (e.g., Oohusa 1980; Makarov et al. 1995; Titlyanov et al. 1996; Kuwano et al. 2008).

We found that low concentrations of APM, griseofulvin and oryzalin arrest mitosis in an ulvophycean alga, *M. angicava* (Fig. 3). APM seems to be the most effective among the three drugs. The lack of dividing cells observed under the high-concentration treatments also supports the idea that these drugs work to depolymerize microtubules in the gametophyte cells of *M. angicava*. This alga appears to have the spindle assembly checkpoint, a mechanism that is important for normal mitosis, as confirmed in land plants (Komaki and Schnittger 2016) because the dividing cells were arrested at metaphase. Our results also indicate that colchicine and 8-hydroxyquinoline are ineffective in *M. angicava*. In two other multinuclear unicellular Ulvophyceae (*Ventricaria ventricosa* and *Valoniopsis pachynema*), McNaughton and Goff (1990) reported that APM inhibits microtubule polymerization, whereas colchicine and griseofulvin appear to be ineffective, although they did not refer to metaphase arrest. APM might also arrest mitosis in other ulvophycean algae. Using these mitotic inhibitors, we successfully observed the typical process of mitosis in *M. angicava* (Fig. 2).

Our three-dimensional fluorescence microscopy observations were useful for counting the number of chromosomes in the gametophyte cells of *M. angicava*. We used prometaphase cells (Fig. 2d), in which the condensed chromosomes are spaced at distances that are suitable for counting chromosomes. In some other species of Ulvophyceae green algae, the number of chromosomes has been examined (e.g., *Chaetomorpha moniligera*, Yabu 1967; *Ulvaria obscura*, Dube 1967; *Cladophora* spp., Zulkifly et al. 2013). In those previous studies, however, the sample cells were observed two-dimensionally after they were pressed tightly between a glass coverslip and a glass slide. We successfully observed individual chromosomes even if they overlapped (Fig. 4, Additional file 1: Video S1). Finally, we identified the number of chromosomes of *M. angicava* as N = 9.

## Additional file

**Additional file 1: Video S1.** Three-dimensional image of the chromosomes in a somatic cell of the haploid gametophyte of *M. angicava*. Nine chromosomes become loosely assembled. Scale bar = 2 µm.

## Data Availability

The datasets used and/or analyzed during the current study are available from the corresponding author on reasonable request.

## Abbreviations

APM: amiprophos methyl
DAPI: 4′-6-diamidino-2-phenylindole
DSU: Disk-Spinning Unit for Confocal Imaging
PES: Provasoli’s enriched sea-water

## References

[CR1] Aruga H, Motomura T, Ichimura T (1996). Immunofluorescence study of mitosis and cytokinesis in *Acrosiphonia duriuscula* (Acrosiphoniales, Chlorophyta). Phycol Res.

[CR3] Dube MA (1967). On the life history of *Monostroma**fuscum* (Postels et Ruprecht) Wittrock. J Phycol.

[CR4] Evans H, Keary GJ, Tonkinson SM (1957). The use of colchicine as an indicator of mitotic rate in broad bean root meristems. J Genet.

[CR5] Horinouchi Y, Yamaguchi M, Chibana H, Togashi T (2019). Nuclear behavior and roles indicate that Codiolum phase is a sporophyte in *Monostroma angicava* (Ulotrichales, Ulvophyceae). J Phycol.

[CR6] Komaki S, Schnittger A (2016). The spindle checkpoint in plants—a green variation over a conserved theme?. Curr Opin Plant Biol.

[CR7] Kuwano K, Sakurai R, Motozu Y, Kitade Y, Saga N (2008). Diurnal cell division regulated by gating the G1/S transition in Enteromorpha compressa (Chlorophyta). J Phycol.

[CR8] Kuwano K, Abe N, Nishi Y, Seno H, Nishihara GN, Iima M, Zachleder V (2014). Growth and cell cycle of Ulva compressa (Ulvophyceae) under LED illumination. J Phycol.

[CR9] Makarov VN, Schoschina EV, Lüning K (1995). Diurnal and circadian periodicity of mitosis and growth in marine macroalgae. I. Juvenile sporophytes of *Laminariales* (Phaeophyta). Eur J Phycol.

[CR10] McNaughton EE, Goff LJ (1990). The role of microtubules in establishing nuclear spatial patterns in multinucleate green algae. Protoplasma.

[CR11] Mine I, Yuasa K, Uesugi M, Sekida S, Okuda K (2011). Band growth and localization of vesicle exocytosis in the red alga *Antithamnion nipponicum* (Ceramiales). Eur J Phycol.

[CR12] Oohusa T (1980). Diurnal rhythm in the rates of cell division, growth and photosynthesis of* Porphyra yezoensis* (Rhodophyceae) cultured in the laboratory. Bot Mar..

[CR13] Provasoli L (1968) Media and prospects for the cultivation of marine algae. In: Watanabe H, Hattori A (ed) Culture and collection of algae. Proceedings U. S.-Japan Cont. Japanese Society of Plant Physiology, Hakone, pp 63–75.

[CR2] R Core Team (2015) R: a language and environment for statistical computing. Vienna: R Foundation for Statistical Computing. https://www.R-project.org/. Accessed 10 Dec 2015.

[CR14] Sommer A, Hoeftberger M, Hoepflinger MC, Schmalbrock S, Bulychev A, Foissner I (2015). Convoluted plasma membrane domains in the green alga *Chara* are depleted of microtubules and actin filaments. Plant Cell Physiol.

[CR15] Takahashi F, Hishinuma T, Kataoka H (2001). Blue light-induced branching in *Vaucheria*. Requirement of nuclear accumulation in the irradiated region. Plant Cell Physiol.

[CR16] Tatewaki M (1969). Culture studies on the life history of some species of the genus *Monostroma*. Sci Pap Inst Algol Res Fac Sci Hokkaido Univ.

[CR17] Titlyanov EA, Titlyanova TV, Lüning K (1996). Diurnal and circadian periodicity of mitosis and growth in marine macroalgae. II. The green alga *Ulva pseudocurvata*. Eur J Phycol.

[CR18] Togashi T, Cox PA (2001). Tidal-linked synchrony of gamete release in the marine green alga, *Monostroma angicava* Kjellman. J Exp Mar Biol Ecol.

[CR19] Togashi T, Motomura T, Ichimura T, Cox PA (1999). Gametic behavior in a marine green alga, *Monostroma angicava*: an effect of phototaxis on mating efficiency. Sex Plant Reprod.

[CR20] Wanner G, Formanek H, Martin R, Herrmann RG (1991). High resolution scanning electron microscopy of plant chromosomes. Chromosoma.

[CR21] Yabu H (1967). Chromosome count in *Chaetomorpha moniligera* KJELLM. Bull Fac Fish Hokkaido Univ.

[CR22] Zulkifly SB, Graham JM, Young EB, Mayer RJ, Piotrowski MJ, Smith I, Graham LE (2013). The genus *Cladophora* Kützing (Ulvophyceae) as a globally distributed ecological engineer. J Phycol.

